# Diagnostic performance of serum vitamin D metabolites and 14 inorganic elements in early prediction of kidney neoplasm

**DOI:** 10.3389/fonc.2026.1884903

**Published:** 2026-07-22

**Authors:** Chunmei Dai, Bin Zhang, Qiang Deng, Jiafu Feng

**Affiliations:** Department of Clinical Laboratory, Mianyang Central Hospital, School of Medicine, University of Electronic Science and Technology of China, Mianyang, China

**Keywords:** C3-epimer, inorganic elements, kidney neoplasm, renal cell carcinoma, vitamin D

## Abstract

**Objective:**

This study aimed to identify potential biomarkers by profiling serum vitamin D (VitD) metabolites and fourteen trace elements, with the goal of screening kidney neoplasm (KN) before symptom onset.

**Methods:**

The study included 57 renal cell carcinoma (RCC) patients, 43 patients with benign KN (BKN), and 50 healthy controls. Serum levels of VitD metabolites, including 25(OH)D_2_, 25(OH)D_3_, and C3-epi-25(OH)D_3_, were quantified using UPLC-MS/MS. The t-25(OH)D was calculated, and f-25(OH)D level was measured by ELISA. Serum concentrations of 14 inorganic elements, including calcium (Ca), magnesium (Mg), copper (Cu), iron (Fe), zinc (Zn), selenium (Se), manganese (Mn), cobalt (Co), silver (Ag), gallium (Ga), cesium (Cs), lead (Pb), cadmium (Cd), and arsenic (As), were determined by ICP-MS.

**Results:**

RCC and BKN patients exhibited significantly lower serum levels of t-25(OH)D, 25(OH)D_3_, f-25(OH)D, Ca, Mg, Fe, Zn, and Ag compared to healthy controls, while C3-epi-25(OH)D_3_ levels were significantly elevated (*P* < 0.05). Additionally, RCC patients had significantly lower levels of Zn and Se, but higher levels of Ga, than BKN patients (*P* < 0.05). Correlation analysis revealed significant positive associations between Fe and Cs with t-25(OH)D, 25(OH)D_3_, C3-epi, and f-25(OH)D, as well as between Ca and t-25(OH)D and f-25(OH)D (*P* < 0.05). Logistic regression analysis identified f-25(OH)D (OR = 0.762, *P* < 0.040), Fe (OR = 0.943, *P* < 0.028), and Zn (OR = 0.945, *P* = 0.001) as protective factors against KN, while C3-epi-25(OH)D_3_ (OR = 84.974, *P* < 0.001) was a risk factor. Receiver operating characteristic (ROC) curve analysis showed that the combination of C3-epi, f-25(OH)D, and Zn (AUC = 0.895), or C3-epi, Fe, and Zn (AUC = 0.912), did not significantly differ from the four-marker combination of C3-epi, f-25(OH)D, Fe, and Zn (AUC = 0.924). All combined approaches outperformed individual or two-marker assays (all *P* < 0.05).

**Conclusion:**

KN patients exhibit altered VitD metabolites and trace element profiles, with low Zn and Se potentially promoting malignancy. Free-25(OH)D, Fe, and Zn serve as protective factors, while C3-epi predisposes to disease. Combining C3-epi with f-25(OH)D and Zn, or with Fe and Zn, provides strong diagnostic accuracy for KN.

## 1 Introduction

Kidney neoplasm (KN) is a common tumor within the urinary system, categorized based on its pathological characteristics into benign KN (BKN) and malignant KN (MKN). BKN is relatively rare in clinical settings, with renal angiomyolipoma being its predominant form. In contrast, MKN is more prevalent and includes subtypes such as renal cell carcinoma (RCC), renal pelvic carcinoma, nephroblastoma, and others, with RCC being the most common. In recent years, RCC has ranked third in both incidence and mortality among urinary system tumors, contributing to approximately 4% of newly diagnosed malignancies worldwide annually (1–3). In 2022, there were approximately 430, 000 new RCC cases globally (4). RCC is an insidious malignancy with an unclear etiology and often presents with nonspecific symptoms. As a result, most patients are diagnosed at advanced stages, with a high likelihood of local spread and distant metastasis, complicating treatment (5). Early detection and diagnosis of RCC are critical for improving treatment outcomes and survival rates.

Vitamin D (VitD), a fat-soluble vitamin, is biologically inactive in its native form and requires hydroxylation in the liver to produce 25-hydroxyvitamin D [25(OH)D], which becomes biologically active. The metabolites, including 25-hydroxyvitamin D_2_ [25(OH)D_2_] and 25-hydroxyvitamin D_3_ [25(OH)D_3_], together comprise the total 25(OH)D [t-25(OH)D], commonly referred to as 25(OH)D. In infants and certain disease states, epimerization at the C3 position of 25(OH)D produces the C3-epimer (C3-epi). While C3-epi shares the same molecular weight and structural formula as 25(OH)D, their spatial configurations differ, and C3-epi is biologically inactive. Current research indicates that, in addition to regulating calcium and phosphorus absorption and maintaining skeletal homeostasis, VitD plays a significant role in inhibiting tumor cell proliferation, modulating immune function, promoting cell differentiation, inducing tumor cell apoptosis, delaying cognitive decline, and downregulating inflammatory factors (6–9). A meta-analysis (10) demonstrated that high levels of 25(OH)D can reduce the risk of renal cancer by 21%. Another study (11) examined the relationship between geographic location and renal cancer incidence in 175 countries, revealing that lower ultraviolet radiation levels were independently associated with a higher renal cancer risk. A case-control study in the Chinese population also found that circulating 25(OH)D levels were inversely associated with renal cancer risk, even after adjusting for confounders (12). However, previous research (13) has suggested that elevated 25(OH)D levels do not correlate with a reduced renal cancer risk. As such, the precise relationship between 25(OH)D levels and renal cancer risk remains controversial, warranting further rigorously designed prospective studies for validation.

The complex structure of the human body consists of various elements, with different inorganic elements playing pivotal roles in numerous physiological and biochemical processes. These elements act as cofactors for enzymes, participate in hormone and vitamin synthesis, regulate immune function, and influence growth and development. Imbalances in the intake of these inorganic elements—whether excessive, deficient, or insufficient—can disrupt physiological functions and contribute to disease development, including tumors (14–18). In the context of tumors, not only do elemental levels impact tumor incidence, but alterations in the forms of these elements, such as their redox states, may also drive tumor initiation and progression. One study (19) reported significantly elevated blood levels of Cu, Mn, As, Cd, and Pb in patients with RCC, while Se levels were notably lower. Another study (20) linked Zn deficiency to an increased risk of renal cancer development.

Both VitD and trace elements are essential nutrients for maintaining normal metabolic and physiological functions. Under various disease conditions, alterations in VitD and/or trace element levels may occur, and these changes can interact synergistically to promote disease onset and progression. One study (21) demonstrated that VitD can enhance iron availability by downregulating pro-inflammatory cytokines and hepcidin. Another study (22) showed that vitamin D_3_ treatment upregulated the expression of transport proteins for iron and zinc. Interactions—both synergistic and antagonistic—are well known among different elements. For instance, excessive zinc intake can induce copper deficiency, leading to leukopenia, anemia, and immunosuppression. Furthermore, the interaction between iron and copper can generate catalytic properties critical for tumor progression (23). Patients with KN have significantly lower concentrations of cobalt, iron, manganese, and zinc compared to healthy controls, suggesting that dysregulation of elemental levels is associated with the development and progression of KN (24). Other studies have indicated that higher VitD levels may significantly reduce the risk of RCC (25).

This study aims to assess serum VitD metabolites and levels of multiple elements in patients with KN (including RCC and BKN) and healthy controls. It will explore the correlations between these elements and renal function indicators, including urea, creatinine (Cr), uric acid (UA), complement 1q (C1q), cystatin C (CysC), neutrophil gelatinase-associated lipocalin (NGAL), and estimated glomerular filtration rate (eGFR). Additionally, the study seeks to examine changes in VitD metabolites and inorganic elements in the serum of KN patients.

## 2 Materials and methods

### 2.1 Study participants

This study included 57 patients with RCC (RCC group, aged 29–77 years, male/female: 37/20), 43 patients with BKN (BKN group, aged 30–84 years, male/female: 21/22), and 50 healthy individuals (HC group, aged 28–83 years, male/female: 33/17) who visited Mianyang Central Hospital between March 2021 and June 2022.

#### 2.1.1 Inclusion criteria

RCC and BKN groups: i) Age ≥ 18 years; ii) Confirmed diagnosis of renal cancer or benign kidney tumor *via* imaging and pathology; iii) Patients with RCC not previously treated with chemotherapy, radiotherapy, or nephrectomy.

Healthy control group: i) Age ≥ 18 years; ii) Volunteers with normal liver and kidney function indicators, blood analysis, and routine urine test results obtained during the same period at our hospital.

#### 2.1.2 Exclusion criteria

i) Pregnant or breastfeeding women; ii) Individuals with acute kidney disease or acute renal failure; iii) Secondary renal function impairment due to conditions such as diabetic nephropathy, hypertensive nephropathy, polycystic kidney disease, malignancies, hyperthyroidism, hypothyroidism, consciousness disorders, or heart failure; iv) Renal parenchymal masses pathologically confirmed as non-renal tumors; v) Patients with RCC undergoing radiotherapy or immunotherapy; vi) Individuals with liver diseases, connective tissue diseases, hematological disorders, or other primary systemic diseases; vii) Patients who have undergone renal dialysis or nephrectomy; viii) Participants who had not used any health supplements or beverages, particularly those rich in vitamins and trace elements.

### 2.2 Sample collection

Subjects (tumor patients prior to treatment) were required to fast overnight for 8–12 hours. Venous blood (approximately 5 mL) was collected into BD Vacutainer tubes (BD, USA) to the marked scale. Within 0.5–1.0 hours, the samples were centrifuged at 2000 g for 10 minutes to separate the serum. Renal function indicators were analyzed within 2 hours. Serum intended for the detection of VitD metabolites and inorganic elements was aliquoted into 1.5 mL low-adsorption centrifuge tubes and stored at –80 °C until analysis.

### 2.3 Research methods

#### 2.3.1 Kidney function indicator test

Serum levels of urea, Cr, UA, C1q, CysC, and NGAL were measured using a Hitachi LST008 automatic biochemical analyzer. The eGFR was calculated based on serum Cr and CysC concentrations following the 2012 Kidney Disease: Improving Global Outcomes (KDIGO) guidelines (26).

#### 2.3.2 Vitamin D metabolites test

VitD metabolites, including 25(OH)D_2_, 25(OH)D_3_, and C3-epi, were analyzed using a UPLC-MS/MS system consisting of a Jasper™ HPLC system (Shimadzu, Japan) and an AB SCIEX Triple Quad™ 4500MD mass spectrometer (ABI, USA). Mass spectra were generated and data processed using the accompanying Analyst^®^ MD and MultiQuant™ MD software. Free 25(OH)D [f-25(OH)D] levels were measured *via* enzyme-linked immunosorbent assay (ELISA), with colorimetric analysis performed using a Rayto microplate reader.

#### 2.3.3 Inorganic elements test

The concentrations of 14 serum inorganic elements were determined using a 7850 ICP-MS (Agilent, USA). Germanium (Ge) served as the internal standard for Ca, Mg, Zn, Fe, Mn, As, and Ga; yttrium (Y) for Cu and Se; scandium (Sc) for Co; indium (In) for Ag, Cd, and Cs; and bismuth (Bi) for Pb. Sample analysis was conducted only when the correlation coefficient (R^2^) of the standard curve for all elements exceeded 0.99.

### 2.4 Statistical analysis

Statistical analysis was performed using SPSS 26.0 software, and graphs were generated with GraphPad Prism 8.0.1. Data with a normal or near-normal distribution are presented as mean ± standard deviation (x̄ ± SD). Differences between experimental and control groups were analyzed using one-way analysis of variance (ANOVA), with pairwise comparisons performed using the Fisher Least Significant Difference test (LSD-t). Data that did not follow a normal distribution are expressed as the median (interquartile range) [M (P25, P75)]. Differences between groups were analyzed using the Kruskal-Wallis H test, and pairwise comparisons were conducted using the Bonferroni method. Correlations were assessed with Pearson’s bivariate correlation analysis (for normally distributed continuous variables) or Spearman’s bivariate correlation analysis (for skewed distribution variables). Binary logistic regression analysis was applied to evaluate the relationship between inorganic elements, VitD metabolites, and the occurrence of KN. Diagnostic performance was assessed using the receiver operating characteristic (ROC) curve, the area under the curve (AUC) comparisons were performed using DeLong’s test. All statistical tests were two-tailed, and a *P*-value < 0.05 was considered statistically significant.

## 3 Results

### 3.1 Characteristics of participants and level of observed indicators

The clinicopathological characteristics of patients with KN are listed in **Table 1**. The levels of renal function indicators, VitD metabolites, and inorganic elements observed in the subjects are presented in **Table 2**. Significant differences were found among the three groups for renal function indicators (including Cr, eGFR, NGAL, and CysC), VitD metabolites (including t-25(OH)D, 25(OH)D_3_, C3-epi, and f-25(OH)D), and inorganic elements (including Ca, Mg, Fe, Zn, Se, Ga, Ag, and Cs) (all *P* < 0.05). These results indicate that, compared to healthy controls, patients with KN exhibit significant alterations in the serum levels of VitD metabolites and certain inorganic elements.

**Table 1:** Clinical and pathological characteristics of KN patients.

Parameter	RCC(n=57)	BKN(n=43)
Age	54.3 ± 12.0	51.9 ± 12.0
Male/female	37(64.91%)/20(35.09%)	21(48.84%)/22(51.16%)
Subtype		
Clear cell RCC	47(82.46%)	
Papillary RCC	5(8.77%)	
Chromophobe RCC	1(1.75%)	
TFE3 RCC	2(3.51%)	
Unclassified	2(3.51%)	
Pathological types		
Angiomyolipoma		31(72.09%)
Oncocytoma		4(9.30%)
Other		8(18.61%)
TNM stage		
I	29(50.88%)	
II	11(19.30%)	
III	13(22.80%)	
IV	4(7.02%)	
WHO/ISUP grade		
1	16(28.07%)	
2	26(45.61%)	
3	4(7.02%)	
4	1(1.75%)	
No grading	10(17.55%)	
Tumor size		
≥5cm	23(40.35%)	
<5cm	34(59.65%)	
Metastasis status		
No metastasis	53(92.98%)	
Metastasis	4(7.02%)	

**Table 2:** Level of renal function indicators, vitamin D metabolites, and inorganic elements in the participants .

Parameter	RCC(n=57)	BKN(n=43)	HC(n=50)	F/χ^2^, *P*
age	54.3 ± 12.0	51.9 ± 12.0	53.2 ± 13.1	0.483^a^, 0.618
male/female	37/20	21/22	33/17	3.548^b^, 0.170
UA	340.0 ± 102.8	303.3 ± 92.1	334.4 ± 56.0	2.228^a^, 0.112
C1q	191.2 ± 39.2	203.8 ± 45.1	191.0 ± 42.2	1.410^a^, 0.247
Cr	86.6 ± 38.0	71.3 ± 19.4	69.7 ± 13.5	6.523^a^, 0.002
eGFR	72.4(61.1, 87.0)	80.2(67.4, 94.7)	85.7(77.7, 95.5)	12.214^b^, 0.002
NGAL	133.0(95.0, 177.0)	136.0(99.1, 202.7)	88.0(68.5, 104.3)	31.148^b^, <0.001
Urea	5.45(4.51, 6.42)	5.37(4.24, 6.48)	5.01(4.47, 5.97)	1.779^b^, 0.411
CysC	1.08(0.90, 1.27)	0.98(0.83, 1.18)	0.92(0.82, 1.01)	10.835^b^, 0.004
t-25(OH)D	21.66 ± 7.05	22.18 ± 9.01	25.97 ± 6.35	4.756, 0.010
25(OH)D_3_	19.01 ± 6.85	19.18 ± 8.35	22.86 ± 6.61	4.451, 0.013
25(OH)D_2_	1.02 ± 0.64	1.14 ± 0.72	1.21 ± 0.93	0.798, 0.452
C3-epi	1.23 ± 0.33	1.24 ± 0.37	0.96 ± 0.32	10.767, <0.001
f-25(OH)D	6.02 ± 2.32	5.89 ± 2.39	7.35 ± 2.45	5.681, 0.004
Ca	2.10 ± 0.20	2.17 ± 0.24	2.28 ± 0.15	12.042^a^, <0.001
Mg	0.87 ± 0.11	0.89 ± 0.11	0.95 ± 0.08	7.951^a^, 0.001
Cu	17.2 ± 4.1	16.9 ± 4.8	16.4 ± 2.4	0.698^a^, 0.499
Fe	45.4 ± 13.0	44.0 ± 10.0	56.0 ± 7.2	18.992^a^, <0.001
Zn	82.7 ± 15.2	93.0 ± 20.0	108.9 ± 21.9	25.588^a^, <0.001
Se	89.4 ± 14.0	105.4 ± 30.1	112.4 ± 17.3	17.215^a^, <0.001
Co	2.84 ± 0.46	2.81 ± 0.39	2.98 ± 0.55	1.805^a^, 0.168
Ga	1.13 ± 0.16	1.04 ± 0.18	1.04 ± 0.18	4.453^a^, 0.013
Mn	34.9(30.6, 38.7)	34.4(31.7, 37.2)	34.7(32.3, 37.5)	0.643^b^, 0.725
Ag	1.72(1.39, 2.01)	1.90(1.33, 2.18)	2.16(1.87, 2.56)	19.838 ^b^, <0.001
Cs	0.55(0.45, 0.71)	0.65(0.48, 0.80)	0.68(0.60, 0.78)	9.707 ^b^, 0.008
Pb	0.65(0.56, 0.80)	0.61(0.46, 0.89)	0.74(0.61, 0.98)	5.709 ^b^, 0.058
Cd	0.12(0.10, 0.17)	0.12(0.10, 0.14)	0.14(0.12, 0.15)	3.667 ^b^, 0.160
As	13.20(12.53, 13.94)	13.25(12.06, 13.76)	12.93(11.91, 13.54)	5.778 ^b^, 0.056

RCC represents renal cell carcinoma, BKN represents benign kidney neoplasms, HC represents healthy controls. ^a^ represents the F-value; ^b^ represents the χ^2^ value. UA and Cr units: μmol/L; Urea unit: mmol/L; C1q and CysC units: mg/L; NGAL unit: μg/L; eGFR unit: mL/min/1.73 m^2^; t-25(OH)D, 25(OH)D_3_, 25(OH)D_2_, and C3-epi units: ng/mL; f-25(OH)D unit: pg/mL; Ca and Mg units: mmol/L; Cu, Fe, and Zn units: μmol/L; Se, Co, Ga, Mn, Ag, Cs, Pb, Cd, and As units: μg/L.

**Figure 1:**
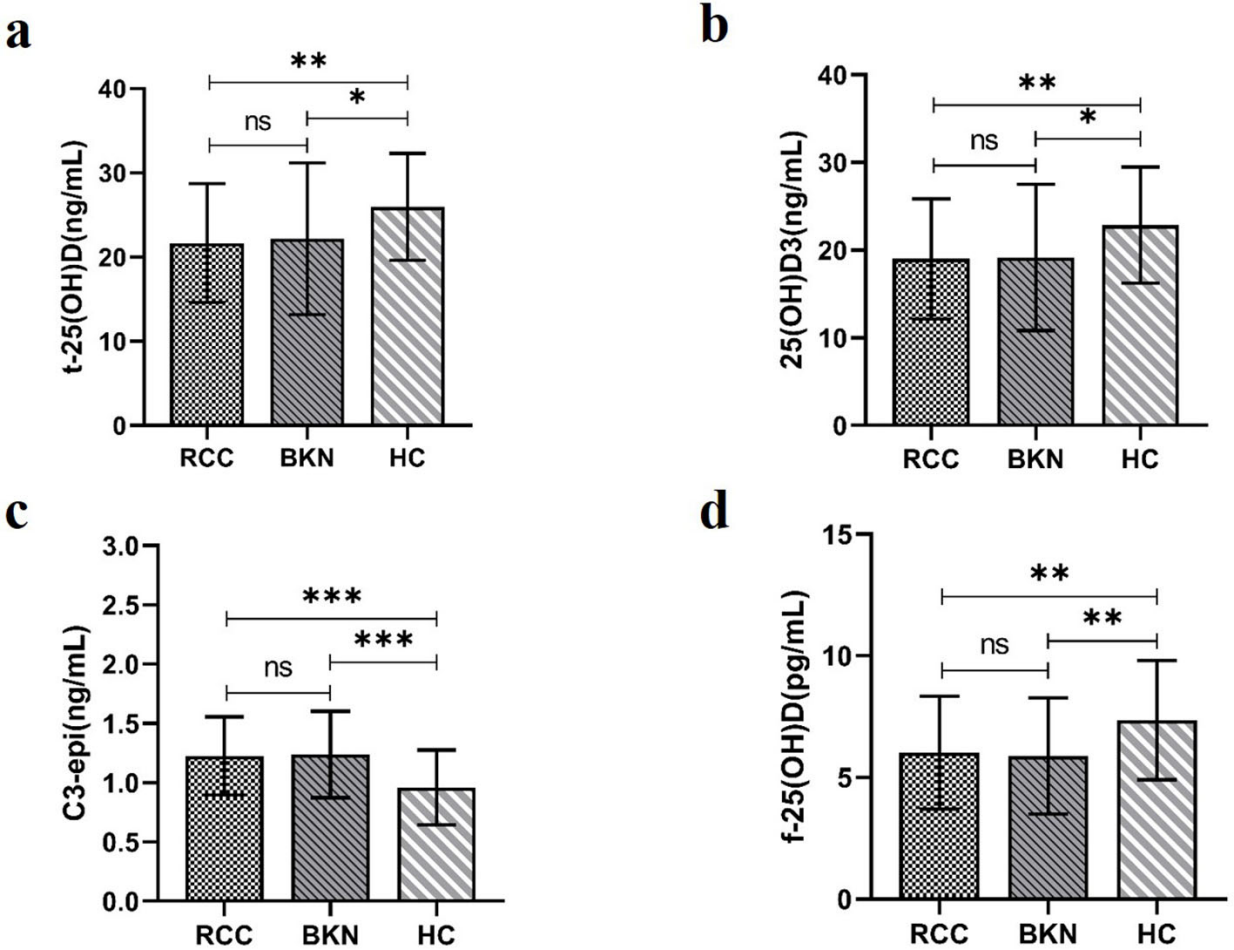
Pairwise comparative analysis of four vitD metabolites in participants. RCC refers to renal cell carcinoma, BKN to benign kidney neoplasms, and HC to healthy controls; * denotes *P* < 0.05, ** denotes *P* < 0.01, *** denotes *P* < 0.001, and ns denotes *P* > 0.05. Four grouped bar charts labeled a to d comparing serum four vitD metabolites among RCC, BKN, and HC groups with statistical significance indicated by asterisks and “ns” for not significant. Chart **(a)** shows t-25(OH)D levels, chart **(b)** shows 25(OH)D3 levels, chart **(c)** shows C3- epi levels, and chart **(d)** shows f-25(OH)D levels, each with error bars and comparisons between groups.

**Figure 2:**
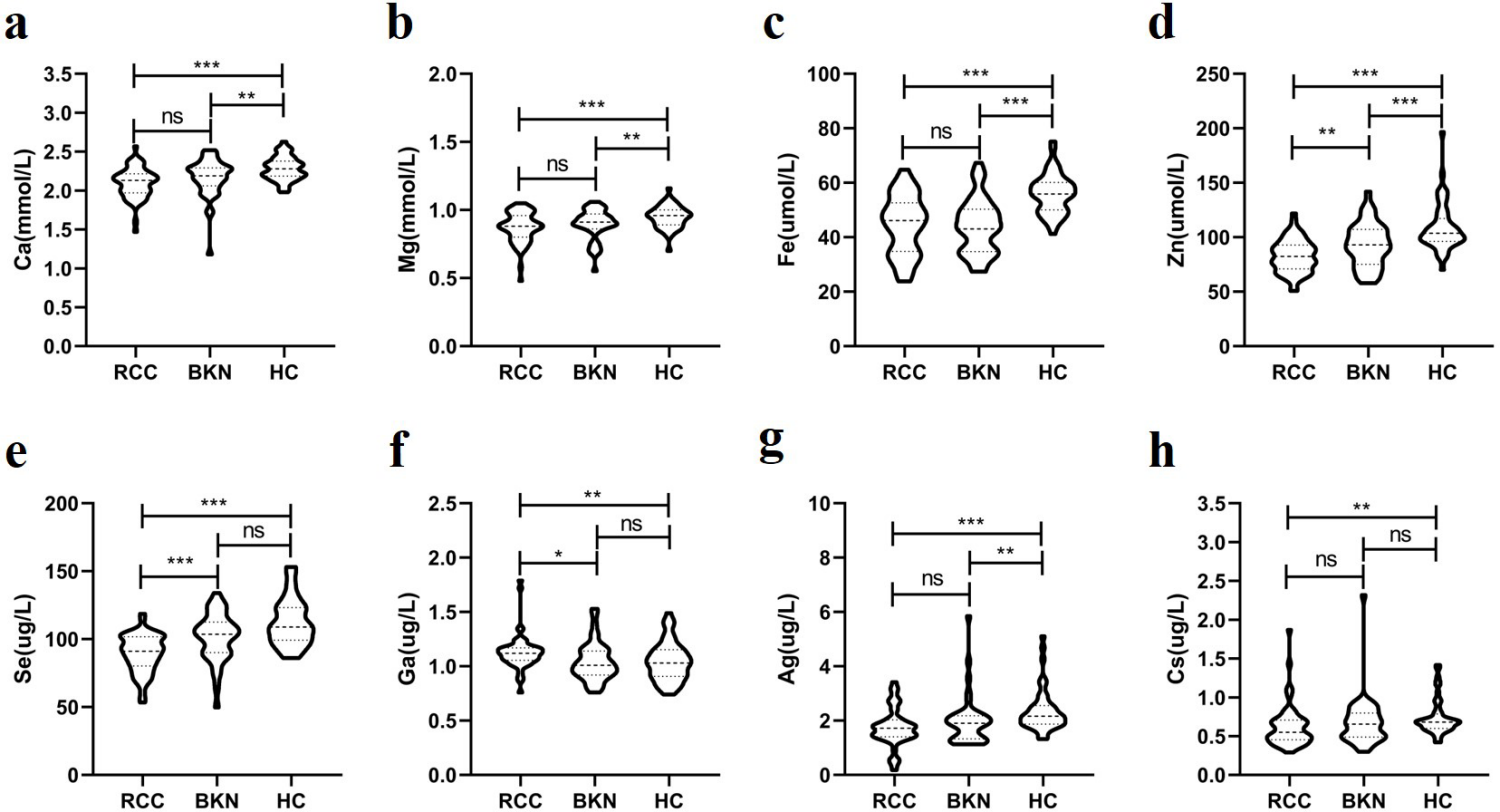
Pairwise comparative analysis of eight elements in participants. RCC refers to renal cell carcinoma, BKN to benign kidney neoplasms, and HC to healthy controls; * denotes *P* < 0.05, ** denotes *P* < 0.01, *** denotes *P* < 0.001, and ns denotes *P* > 0.05. Eight violin plots labeled **(a–h)** compare serum concentrations of Ca, Mg, Fe, Zn, Se, Ga, Ag, and Cs across three groups: RCC, BKN, and HC. Statistical significance is denoted using asterisks above each comparison, where more asterisks indicate greater significance, and “ns” indicates not significant. Data ranges and median values vary for each element and group.

To identify the indicators that differed among the observed groups, pairwise comparisons were performed on three VitD metabolites and eight inorganic elements that exhibited significant intergroup differences. The results revealed that in RCC patients, the levels of t-25(OH)D, 25(OH)D_3_, and f-25(OH)D (**Figures 1a, b, d**), as well as Ca, Mg, Fe, Zn, Se, Ag, and Cs (**Figures 2a–e, g, h**), were significantly lower (all *P* < 0.05), whereas C3-epi and Ga were significantly higher compared to the healthy control group (all *P* < 0.05, **Figures 1c**, **2f**). In BKN patients, the levels of t-25(OH)D, 25(OH)D_3_, and f-25(OH)D (**Figures 1a, b, d**), as well as Ca, Mg, Fe, Zn, and Ag (**Figures 2a–d, g**), were significantly lower than those in healthy controls (all *P* < 0.05), while C3-epi was significantly elevated (*P* < 0.05, **Figure 1c**). When compared to BKN patients, RCC patients exhibited significantly lower levels of Zn (*P* < 0.05, **Figure 2d**) and Se (*P* < 0.05, **Figure 2e**), and significantly higher levels of Ga (*P* < 0.05, **Figure 2f**). These results suggest that both RCC and BKN patients show alterations in VitD metabolites and inorganic elements compared to healthy controls. However, aside from the significant differences in Zn, Se, and Ga levels between RCC and BKN patients, no substantial deviations were noted for the other indicators.

### 3.2 Correlation between observed indicators in patients with kidney neoplasms

**Figure 3:**
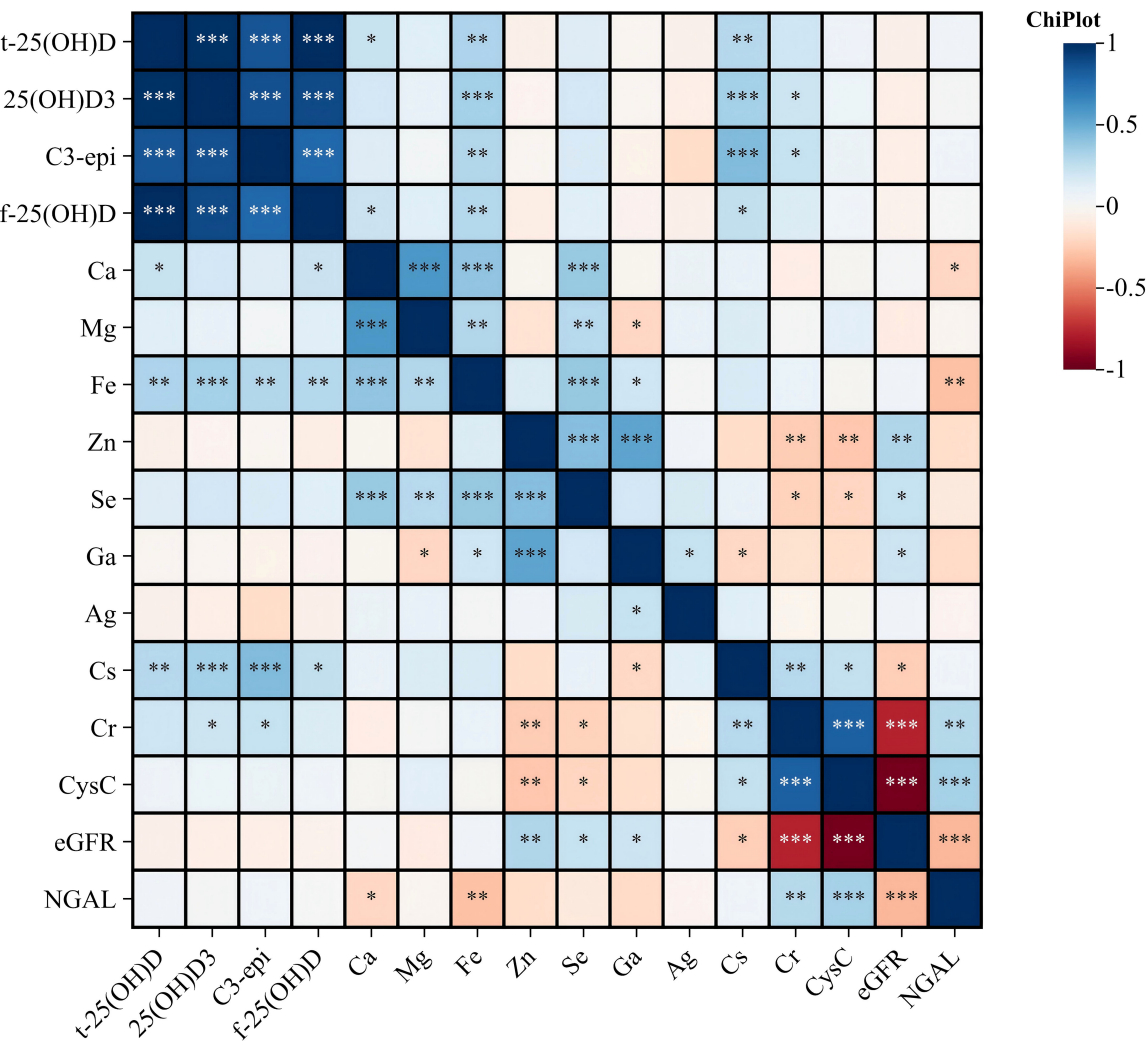
Correlation between serum vitD metabolites, inorganic elements, and renal function indicators in KN patients. * denotes *P* < 0.05, ** denotes *P* < 0.01, *** denotes *P* < 0.001, and ns denotes *P* > 0.05. ChiPlot uses colors from dark blue to dark red to represent correlation coefficients (r) ranging from -1 to +1.

To explore the relationship between VitD metabolites, inorganic elements, and renal function indicators in KN patients, correlation analysis was performed on 12 indicators showing differences among RCC, BKN, and HC groups. The results revealed that t-25(OH)D was significantly positively correlated with Ca (r = 0.225, *P* = 0.028), Fe (r = 0.322, *P* = 0.001), and Cs (r = 0.294, *P* = 0.004) (**Figure 3**); 25(OH)D_3_ was significantly positively correlated with Fe (r = 0.343, *P* < 0.001), Cs (r = 0.344, *P* < 0.001), and Cr (r = 0.216, *P* = 0.031) (**Figure 3**); C3-epi showed a significant positive correlation with Fe (r = 0.303, *P* = 0.002), Cs (r = 0.440, *P* < 0.001), and Cr (r = 0.241, *P* = 0.016) (**Figure 3**); f-25(OH)D was significantly positively correlated with Ca (r = 0.221, *P* = 0.027), Fe (r = 0.294, *P* = 0.003), and Cs (r = 0.254, *P* = 0.011) (**Figure 3**); Ca was significantly negatively correlated with NGAL (r = -0.211, *P* = 0.035) (**Figure 3**); Fe was significantly negatively correlated with NGAL (r = -0.299, *P* = 0.003) (**Figure 3**); Zn was significantly positively correlated with eGFR (r = 0.308, *P* = 0.002) and negatively correlated with Cr (r = -0.258, *P* = 0.010) and CysC (r = -0.280, *P* = 0.005) (**Figure 3**); Se showed a significant positive correlation with eGFR (r = 0.243, *P* = 0.015) and a negative correlation with Cr (r = -0.228, *P* = 0.022) and CysC (r = -0.214, *P* = 0.032) (**Figure 3**); Ga was significantly positively correlated with eGFR (r = 0.212, *P* = 0.034) (**Figure 3**); Cs was significantly positively correlated with Cr (r = 0.287, *P* = 0.004) and CysC (r = 0.250, *P* = 0.012), and negatively correlated with eGFR (r = -0.247, *P* = 0.013) (**Figure 3**). These results suggest that changes in VitD metabolites and inorganic elements in KN patients may be linked to renal function alterations. Furthermore, correlations between VitD metabolites and inorganic elements indicate that their interactions could influence changes in renal function.

### 3.3 Vitamin D metabolites and inorganic elements in KN occurrence

To further investigate the relationship between VitD metabolites, inorganic elements, and KN occurrence, multivariate logistic regression analysis was performed on the abnormally altered VitD metabolites and inorganic elements observed in KN patients. Additionally, eGFR was included as a covariate to adjust the results (**Table 3**). After adjusting for the effect of eGFR, the results showed that in predicting KN occurrence, C3-epi, f-25(OH)D, Fe, and Zn exhibited significant differences (all *P* < 0.05), with f-25(OH)D, Fe, and Zn acting as protective factors and C3-epi as a risk factor. In predicting RCC occurrence, C3-epi, Zn, and Se showed significant differences (all *P* < 0.05), with Zn and Se serving as protective factors and C3-epi as a risk factor. For BKN occurrence prediction, C3-epi, Fe, and Zn demonstrated significant differences (all *P* < 0.05), with Fe and Zn acting as protective factors and C3-epi as a risk factor. These results indicate that among the 14 elements and 4 VitD metabolites, Fe, Zn, f-25(OH)D, and C3-epi are associated with KN occurrence. Of these, Fe, Zn, and f-25(OH)D are protective factors for KN/RCC occurrence, while C3-epi serves as a risk factor.

**Table 3:** Logistic regression analysis of serum vitamin D metabolites and inorganic elements in KN patients.

Variable	KN				RCC				BKN			
	Unadjusted		Adjusted*		Unadjusted		Adjusted*		Unadjusted		Adjusted*	
	*P*	OR (95%CI)	*P*	OR (95%CI)	*P*	OR (95%CI)	*P*	OR (95%CI)	*P*	OR (95%CI)	*P*	OR (95%CI)
25(OH)D_3_	0.156	0.922 (0.824, 1.032)	0.115	0.909 (0.808, 1.023)	0.073	0.926 (0.852, 1.007)	0.056	0.920 (0.845, 1.002)	0.153	0.885 (0.749, 1.047)	0.132	0.879 (0.743, 1.039)
C3-epi	<0.001	84.974 (7.230, 998.728)	0.001	97.806 (7.374, 1297.230)	0.004	8.298 (1.930, 35.681)	0.011	7.246 (1.583, 33.158)	0.001	159.214 (8.701, 2913.404)	0.001	161.601 (8.787, 2971.975)
f-25(OH)D	0.040	0.762 (0.587, 0.988)	0.049	0.769 (0.591, 0.999)	0.522	0.931 (0.748, 1.159)	0.642	0.950 (0.763, 1.182)	0.241	0.754 (0.471, 1.208)	0.243	0.758 (0.476, 1.207)
Ca	0.256	0.112 (0.003, 4.876)	0.222	0.088 (0.002, 4.352)	0.206	0.142 (0.007, 2.930)	0.142	0.097 (0.004, 2.184)	0.868	1.404 (0.026, 75.909)	0.832	1.543 (0.028, 85.469)
Mg	0.525	0.065 (0.000, 299.879)	0.593	0.094 (0.000, 552.233)	0.624	0.262 (0.001, 55.629)	0.516	0.143 (0.000, 50.932)	0.599	0.127 (0.000, 278.607)	0.656	0.176 (0.000, 370.606)
Fe	0.028	0.943 (0.896, 0.994)	0.047	0.949 (0.901, 0.998)	0.333	1.019 (0.981, 1.058)	0.117	1.034 (0.992, 1.077)	<0.001	0.861 (0.796, 0.931)	<0.001	0.855 (0.788, 0.927)
Zn	0.001	0.945 (0.916, 0.976)	0.006	0.954 (0.923, 0.987)	0.002	0.954 (0.926, 0.984)	0.012	0.962 (0.933, 0.991)	0.020	0.964 (0.935, 0.994)	0.016	0.960 (0.929, 0.992)
Se	0.458	0.990 (0.963, 1.017)	0.360	0.987 (0.960, 1.015)	0.020	0.961 (0.928, 0.994)	0.015	0.959 (0.928, 0.992)	—	—	—	—
Ag	0.267	1.037 (0.973, 1.105)	0.415	1.069 (0.911, 1.254)	0.950	0.998 (0.953, 1.046)	0.708	1.010 (0.960, 1.062)	0.933	0.994 (0.865, 1.143)	0.891	0.991 (0.868, 1.131)

OR, odds ratio; *Adjusted by eGFR.

### 3.4 Diagnostic performance of vitamin D metabolites and inorganic elements in predicting KN occurrence

**Figure 4:**
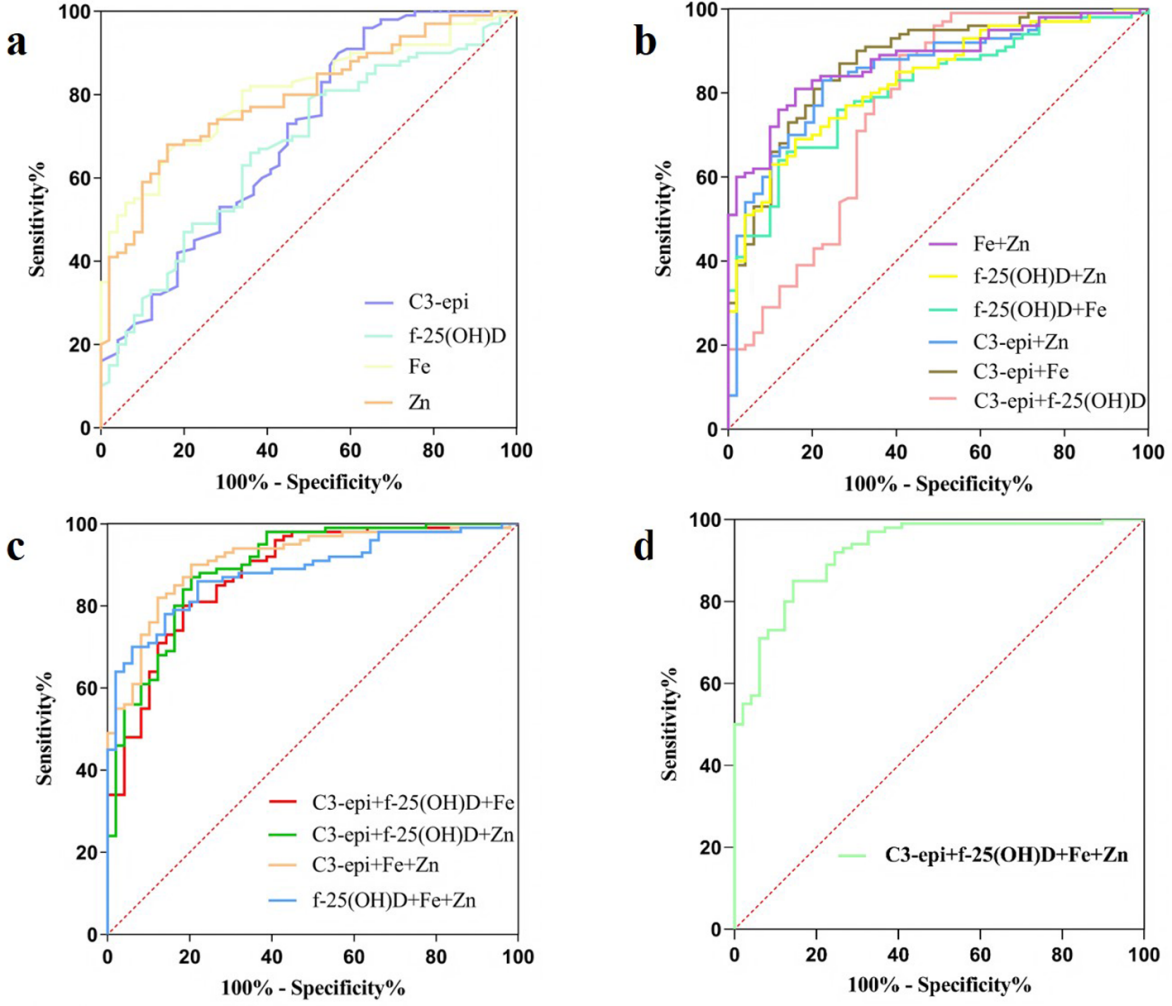
ROC curves of the four differential indicators in KN patients. **(a)** Individual detection; **(b)** Two-marker panel detection; **(c)** Three-marker panel detection; **(d)** Four-marker panel detection.

To analyze the diagnostic performance of VitD metabolites and inorganic elements in predicting KN occurrence, ROC analysis was conducted on four indicators (**Table 4**; **Figure 4**). The four indicators and combined-marker panels were generated using multivariate logistic regression analysis, with the disease status as the dependent variable and the candidate biomarkers as independent variables. Diagnostic efficacy was evaluated by the area under the curve (AUC). The cutoff values for C3-epi, f-25(OH)D, Fe, and Zn, determined based on the maximum Youden index (YI), were 0.87 ng/mL, 6.68 pg/mL, 48.70 μmol/L, and 94.45 μmol/L, respectively (**Table 4**).

**Table 4:** Analysis of the diagnostic efficacy of four differential indicators for KN occurrence.

Indicator	AUC (95% CI)	Se(%)	Sp(%)	YI
Individual detection				
C3-epi	0.703(0.611, 0.794)	90.0	42.9	0.329
f-25(OH)D	0.674(0.585, 0.763)	66.0	64.0	0.300
Fe	0.804(0.735, 0.872)	66.0	84.0	0.500
Zn	0.794(0.723, 0.865)	68.0	84.0	0.520
Two-Marker panel				
C3-epi+f-25(OH)D	0.764(0.675, 0.852)	89.0	59.2	0.482
C3-epi+Fe	0.872(0.813, 0.932)	81.0	79.6	0.606
C3-epi+Zn	0.851(0.787, 0.915)	83.0	77.6	0.606
f-25(OH)D+Fe	0.811(0.742, 0.879)	66.0	86.0	0.520
f-25(OH)D+Zn	0.830(0.765, 0.895)	69.0	84.0	0.530
Fe+Zn	0.877(0.823, 0.931)	81.0	84.0	0.650
Three-Marker panel				
C3-epi+f-25(OH)D+Fe	0.878(0.820, 0.937)	80.0	81.6	0.616
C3-epi+f-25(OH)D+Zn	0.895(0.841, 0.950)	87.0	79.6	0.666
C3-epi+Fe+Zn	0.912(0.865, 0.959) ^▲^	82.0	87.8	0.698
f-25(OH)D+Fe+Zn	0.884(0.832, 0.936)	70.0	94.0	0.640
Four-Marker panel				
C3-epi+f-25(OH)D+Fe+Zn	0.924(0.881, 0.967) ^*, #^	85.0	85.7	0.707

AUC, area under the ROC curve; Se, sensitivity; Sp, specificity; YI = Se + Sp - 1. ^*^indicates comparison with “C3-epi+Fe+Zn”, z=1.153, *P* = 0.249; ^#^indicates comparison with “C3-epi+f-25(OH)D+Zn”, z=1.789, *P* = 0.074; ^▲^indicates comparison with “Fe+Zn”, z=2.014, *P* = 0.044.

When tested individually, C3-epi exhibited the highest sensitivity (Se) of 90.0%, but its diagnostic performance was relatively modest, with an AUC of 0.703 and a YI of 0.327 (**Table 4**). Fe and Zn showed the best specificity (Sp) of 84.0% and demonstrated superior diagnostic performance (with no significant difference between the two), achieving AUC values of 0.804 and 0.794, respectively. However, their YI values were also relatively low, with the maximum YI for Fe being 0.520 (**Table 4**). Based on these findings, combined detection was further explored to assess the diagnostic performance of the observed indicators. Combined analysis revealed that the four-marker panel (C3-epi+f-25(OH)D+Fe+Zn) achieved the highest diagnostic performance (AUC = 0.924), though it showed no statistical difference compared to the three-marker panel (C3-epi+Fe+Zn, z = 1.153, *P* = 0.249) and (C3-epi+f-25(OH)D+Zn, z = 1.789, *P* = 0.074), indicating similar diagnostic performance. There were no statistical differences between the various three-marker panels (all *P* > 0.05). Among these, the C3-epi+Fe+Zn panel was significantly superior to the two-marker panel (Fe+Zn), which had the highest AUC among the two-marker combinations (z = 2.014, *P* = 0.044).

These results suggest that combined detection can significantly enhance the diagnostic performance of the observed indicators in predicting KN occurrence. The three-marker panel (C3-epi+f-25(OH)D+Zn or C3-epi+Fe+Zn) provided the highest diagnostic performance, comparable to the four-marker panel (C3-epi+f-25(OH)D+Fe+Zn).

## 4 Discussion

Vitamins and trace elements are essential nutrients necessary for human growth, material metabolism, and the maintenance of normal physiological functions. As a fat-soluble vitamin, the metabolic homeostasis of VitD, along with the balance of various inorganic elements (such as Ca, Fe, Zn, etc.), forms a core network for the body’s material metabolism and immune regulation. Disruptions in VitD and inorganic elements have been confirmed to be closely associated with the occurrence and progression of various solid tumors (27). Serum Cu levels are significantly elevated in breast cancer patients, while Zn and Se levels are significantly reduced (28). In prostate cancer patients, blood levels of Se, Zn, and Mn are significantly decreased, whereas Fe and Cu levels are significantly increased (29, 30). In RCC patients, the levels of Ca, Cd, Mg, Mn, Pb, and Sr in renal tumor tissues are markedly altered, suggesting that these elements may play pivotal roles in the development and progression of RCC (31).

This study employed precise detection techniques, such as UPLC-MS/MS and ICP-MS, to analyze the serum levels of VitD metabolites and 14 inorganic elements in KN patients (including RCC and BKN). By integrating renal function indicators and statistical models, the research further elucidated the roles of VitD and inorganic elements in the occurrence of KN, as well as their clinical diagnostic value. The biological effects of VitD depend on the precise regulation of its active metabolites. Total-25(OH)D, which is the sum of 25(OH)D_2_ and 25(OH)D_3_, is the gold standard for assessing VitD status in the human body. Meanwhile, f-25(OH)D, the active form capable of directly penetrating cell membranes, serves as a key molecule mediating the anti-inflammatory and anti-tumor effects of VitD (32). In the present study, serum levels of t-25(OH)D, 25(OH)D_3_, and f-25(OH)D in KN patients were significantly lower than those in healthy controls, while C3-epi levels were significantly elevated. This confirms that VitD metabolic abnormalities are associated with KN occurrence (10, 12). However, no significant changes were observed in 25(OH)D_2_ in KN patients, likely due to its much lower concentration in peripheral blood compared to 25(OH)D_3_. Additionally, because of its low abundance, its C3-epimer was not detected, making it impossible to assess whether it undergoes epimerization.

Inorganic elements act as cofactors for enzymes and signaling molecules in cellular metabolism. Their imbalance can influence tumor progression through mechanisms such as oxidative stress and immune suppression (14, 18). In the present study, serum levels of Ca, Mg, Fe, Zn, Se, Ag, and Cs in KN patients were significantly lower than in healthy participants, while Ga levels were significantly higher. Notably, Zn, Se, and Ga levels differed between benign and malignant tumor groups, suggesting that changes in the concentrations of inorganic elements may be linked to the onset of KN. Previous research (24) has confirmed that decreased levels of multiple elements, including Co, Cr, Fe, Mn, Ni, and Zn, may contribute to the development of KN. Elevated blood levels of As, Cu, Mn, Cd, and Pb have been found to be potentially associated with RCC occurrence (19). Although the types and levels of elements reported vary across studies, they collectively suggest that alterations in inorganic elements may lead to KN onset. Discrepancies in research findings may be influenced by differences in study populations, as variations in occupation, diet, and environment can lead to divergent results. Furthermore, factors such as differences in sample size and source may not always capture the association between heavy metal elements and KN. Additionally, variations in detection methods for inorganic elements and potential metabolic differences among individuals could contribute to inconsistent findings, with possible cumulative effects on kidney damage.

Zinc (Zn) is a key component of numerous proteins and regulates various cellular and subcellular functions. Zn deficiency can exacerbate the progression of chronic kidney disease (CKD), and as CKD advances to end-stage, Zn is increasingly eliminated from the body, further worsening the deficiency (24, 33). This study found that Zn depletion was more severe in RCC patients compared to those with BKN. Se, a component of several antioxidant enzymes such as glutathione peroxidase, superoxide dismutase, and thioredoxin reductase (34), plays a vital role in the body’s immune function. One study has reported (35) that Se offers protective effects on the kidneys, mitigating toxin-induced damage. Conversely, Se deficiency may promote kidney disease progression and even shorten the survival of renal cancer patients. Gallium (Ga), a non-essential trace element in humans, is frequently used in positron emission tomography (PET) for tumor imaging and anti-tumor therapy (36). However, no literature has yet reported its association with the occurrence or progression of KN. Ag is a non-essential trace element, and current literature has not established an association between its low circulating concentrations and the etiology of kidney nephropathy (KN). Existing research has predominantly focused on the applications of silver nanoparticles in antitumor therapy (37). Although statistically significant intergroup differences in serum Ga and Ag concentrations were observed in the present study, absolute levels remained consistently low across all three cohorts. Notably, statistically significant intergroup differences were observed for Ga, Cs, and Ag, trace elements detected at extremely low serum concentrations. In the absence of mechanistic validation, the biological relevance of these findings remains to be fully elucidated. Furthermore, multiple statistical testing carries an inherent risk of spurious correlations, mandating cautious interpretation of the above associations. This observed difference may be attributed to the small sample size or sampling variations. By analyzing the correlation between differential VitD metabolites, inorganic elements, and renal function indicators, significant correlations were found between 25(OH)D_3_, f-25(OH)D, Fe, Zn, Se, and certain renal function indicators. This suggests that fluctuations in their levels may influence renal function changes in KN patients. Since the kidneys play a critical role in maintaining normal levels of VitD and various elements, it is also possible that alterations in renal function lead to shifts in these substances, further aggravating the disease. Furthermore, its cross-sectional observational design only identifies associations and excludes causal inference, leaving unresolved whether altered vitamin D metabolites and trace elements promote kidney lesion pathogenesis or represent secondary outcomes of the disease and renal impairment. Additionally, C3-epi was identified as a risk factor for KN occurrence and showed significant correlations with several renal function indicators, implying that elevated C3-epi levels may contribute to renal function changes and increase the risk of KN.

The kidney is responsible for activating VitD and serves as a target organ for many inorganic elements. In KN patients, renal function is already impaired, and imbalances or abnormalities in VitD and inorganic elements may exacerbate kidney injury. The correlation analysis in this study revealed that changes in serum Fe levels were associated with 25(OH)D_3_, C3-epi, and f-25(OH)D, suggesting potential interactions between Fe and VitD metabolites. Fe deficiency may reduce the synthesis of 25(OH)D_3_ and f-25(OH)D, indicating that decreased Fe levels could contribute to VitD deficiency. This is likely because Fe is involved in the activation of VitD hydroxylases, thereby influencing VitD metabolism (38). Fe deficiency may also affect the conversion of 25(OH)D_3_ (39), leading to VitD deficiency. Another study (40) reported that the impact of Fe deficiency on VitD status is more pronounced than the effect of VitD levels on Fe status. The second step in the activation of 25(OH)D_3_ occurs in the kidneys, dependent on cytochrome P450 enzymes. Fe deficiency impairs cytochrome P450 activity, hindering the activation of 25(OH)D_3_ and potentially promoting the development of KN (9).

This study also found that Ca levels in KN patients were associated with both f-25(OH)D and t-25(OH)D levels, reinforcing the role of VitD in Ca absorption and confirming its well-established function in promoting intestinal Ca uptake (27, 41). These findings suggest that Ca imbalance may contribute to the development and progression of KN (42). Cs, a radioactive element, is typically present in very low levels in the human body. Prolonged exposure to Cs-containing environments can elevate its levels and potentially lead to disease. In this study, Cs levels in all three groups were very low, precluding the establishment of a causal relationship between Cs and KN occurrence. Future research should employ animal models to validate the relationship between Cs and KN and further investigate the potential link between Cs and VitD metabolites.

To further elucidate the relationship between VitD metabolites, inorganic elements, and the occurrence of KN, a multifactorial logistic regression analysis was conducted on the abnormally altered VitD metabolites and inorganic elements in KN patients. The analysis revealed that a decrease in f-25(OH)D, Fe, and Zn levels, coupled with an increase in C3-epi levels, could elevate the risk of KN. Similarly, reduced Zn and Se levels, combined with an increase in C3-epi, could raise the risk of RCC, while a decline in Fe and Zn levels, alongside an increase in C3-epi, could heighten the risk of BKN. f-25(OH)D, which can freely cross the membrane of renal proximal tubular epithelial cells and undergo hydroxylation, is the VitD metabolite responsible for exerting physiological functions in the body (32). Higher circulating VitD levels are associated with a reduced risk of RCC (43). C3-epi, an isomer of 25(OH)D_3_ with no biological activity, increases as 25(OH)D_3_ levels decrease, thereby raising the risk of RCC. Previous research (22) has indicated that treatment with 25(OH)D_3_ significantly upregulates genes involved in iron homeostasis, such as ceruloplasmin, haptoglobin, and SLC46A1, while also increasing the expression of the zinc transporter SLC30A10, which elevates Fe and Zn levels in the blood. If the isomerization of 25(OH)D_3_ to C3-epi increases, the storage of active VitD decreases, potentially leading to reduced Fe and Zn levels in the blood. This could trigger various oxidative stress responses in the body, contributing to KN development. Various heavy metal elements, such as Pb, Cd, and As, can cause kidney injury, with long-term exposure potentially leading to CKD (44, 45). However, this study did not find a significant association between heavy metal elements and the occurrence of KN, which may be due to sampling variability or the living environments of the participants.

The early diagnosis of KN, particularly RCC, predominantly relies on imaging techniques such as ultrasound, CT, MRI, and renal biopsy. However, these methods have limitations and risks, including a high rate of missed diagnoses (with detection rates for tumors < 1 cm in diameter being < 30%) and invasiveness (needle biopsy), leading to poor patient compliance (5). To explore reliable laboratory-assisted diagnostic tools for clinical practice, the diagnostic performance of f-25(OH)D, C3-epi, Fe, and Zn was evaluated in KN patients. The results revealed that the diagnostic performance of each individual marker was moderate, but combined detection significantly improved diagnostic accuracy. Notably, the combined detection of either C3-epi, f-25(OH)D, and Zn or C3-epi, Fe, and Zn achieved diagnostic performance comparable to the four-marker combination. For high-risk KN patients, stratification using a three-marker combination test is recommended. Those with abnormalities in all three markers should undergo imaging and renal biopsy, facilitating an “early detection, early intervention” approach to KN prevention and management. To mitigate overfitting risks arising from limited event numbers across RCC and BKN subgroups and the inclusion of multiple predictors, rigorous feature selection incorporating binary logistic regression was implemented with strict adherence to the Events Per Variable (EPV) criterion. This strategy served to constrain model complexity and stabilize odds ratio estimates, thereby avoiding excessively wide confidence intervals. Although the absence of an independent external validation cohort suggests that the reported AUC values may exhibit optimistic bias and might not fully replicate in routine clinical settings, internal validation outcomes indicate that the model captures biologically meaningful signals rather than random noise.

## 5 Conclusion

This study measured serum VitD metabolites and 14 inorganic elements in KN patients and healthy controls, conducting correlation analyses and diagnostic performance evaluations. The findings suggest that alterations in the levels of certain VitD metabolites and inorganic elements may contribute to the occurrence of KN and renal dysfunction. Specifically, reduced levels of Zn and Se may promote the malignant transformation of renal tumors. f-25(OH)D, Fe, and Zn were identified as protective factors against KN, while C3-epi was found to be a risk factor. The combined detection of C3-epi, f-25(OH)D, and Zn, or C3-epi, Fe, and Zn, demonstrated good diagnostic performance in predicting the occurrence of KN, offering a novel approach for early clinical screening. Nevertheless, these preliminary findings should be interpreted with caution due to the modest single-center sample size, which limits statistical power, generalizability and external validity. Additionally, lifestyle-related covariates including dietary patterns, nutritional supplements, and socioeconomic status can substantially interfere with the evaluation of vitamin D metabolic profiles. Future research should involve multicenter, large-sample prospective studies, combined with animal and cellular experiments, to further investigate the molecular mechanisms through which VitD metabolites and inorganic elements influence the development and progression of renal tumors. This would help validate the diagnostic efficacy and clinical utility of the combined detection approach, providing stronger scientific evidence for the prevention, early diagnosis, and treatment of KN.

## Data Availability

The original contributions presented in the study are included in the article/supplementary material. Further inquiries can be directed to the corresponding author.
